# Enhancing Racial/Ethnic Equity in College Student Mental Health Through Innovative Screening and Treatment

**DOI:** 10.1007/s10488-021-01163-1

**Published:** 2021-09-09

**Authors:** Tamar Kodish, Anna S. Lau, Elizabeth Gong-Guy, Eliza Congdon, Inna Arnaudova, Madison Schmidt, Lauren Shoemaker, Michelle G. Craske

**Affiliations:** 1 Department of Psychology, University of California, Los Angeles, 502 Portola Plaza, Franz Hall 1285, Los Angeles, CA 90095-1563 USA; 2 Jane and Terry Semel Institute for Neuroscience and Human Behavior, University of California, Los Angeles, 760 Westwood Plaza, Los Angeles, CA 90095 USA; 3 Department of Psychology, Northwestern University, 2029 Sheridan Road, Swift Hall 102, Evanston, IL 60208 USA; 4 Department of Psychiatry and Biobehavioral Sciences, University of California, Los Angeles, 760 Westwood Plaza, Los Angeles, CA 90095 USA

## Abstract

Although college campuses are diversifying rapidly, students of color remain an underserved and understudied group. Online screening and subsequent allocation to treatment represents a pathway to enhancing equity in college student mental health. The purpose of the current study was to evaluate racial/ethnic differences in mental health problems and treatment enrollment within the context of a largescale screening and treatment research initiative on a diverse college campus. The sample was comprised of n = 2090 college students who completed an online mental health screening survey and were offered either free online or face-to-face treatment based on symptom severity as a part of a research study. A series of ordinal, binomial and multinomial logistic regression models were specified to examine racial/ethnic differences in mental health problems, prior treatment receipt, and enrollment in online and face-to-face treatment through the campus-wide research initiative. Racial/ethnic differences in depression, anxiety and suicidality endorsed in the screening survey were identified. Students of color were less likely to have received prior mental health treatment compared to non-Hispanic white students, but were equally likely to enroll in and initiate online and face-to-face treatment offered through the current research initiative. Rates of enrollment in online therapy were comparable to prior studies. Online screening and treatment may be an effective avenue to reaching underserved students of color with mental health needs on college campuses. Digital mental health tools hold significant promise for bridging gaps in care, but efforts to improve uptake and engagement are needed.

## Introduction

Mental illness among college students is a public health crisis, with rates of depression and anxiety more than doubling over the past decade. In 2019, 42.2% of U.S. college students reported feeling so depressed it was difficult to function, and 63.6% of college students reported experiencing overwhelming anxiety (Duffy et al., 2019). Almost one in four college students have experienced suicidal ideation in their lifetime (Mortier et al., 2018). In the face of exploding mental health need on college campuses, counseling centers have observed large increases in treatment seeking (Xiao et al., 2017). Despite efforts to respond to increased demand, many campuses lack sufficient resources to support student mental health needs (Watkins et al., 2012). This shortage, compounded by a myriad of barriers to mental health services (e.g. stigma, limited financial resources, lack of time), has left the majority of college students suffering from mental health concerns without treatment (Downs & Eisenberg, 2012; Miranda et al., 2015). Improved understanding of student mental health needs and patterns of service use on diverse college campuses is needed to begin bridging gaps in unmet need for care.

To date, the majority of our knowledge about mental health problems and treatment on college campuses is derived primarily from non-Hispanic white (NHW) samples of students. For example, two of the largest nationally conducted studies, the National College Health Assessment (NCHA), and the Healthy Minds Study (HMS), were composed of 72% and 74% NHW students, respectively (Duffy et al., 2019). Despite this representation in recent studies, NHW college students currently comprise just 54.8% of the U.S. college student population (Espinosa et al., 2019). Although the extant literature includes predominantly NHW college student samples, the percentage of American college students of color is increasing, whereas the proportion of NHW students is decreasing. From 1976 to 2016, the percentage of Latinx students in the U.S. postsecondary student population grew from 4 to 18%, and the percentage of Black students increased from 10 to 14%. Asian American enrollment also increased more than threefold within this timespan. Across this time, the percentage of NHW college students fell from 84 to 57% (U.S. Department of Education, 2018). Given these demographic shifts, increased attention to the mental health and service use of racial/ethnic minority college students is needed.

Currently, less is known about mental health problems and service use among college students of color relative to their non-Hispanic white counterparts. Some data suggest that students from some racial/ethnic minority groups experience elevated depression, anxiety and suicide risk when compared to NHW students or all other students in college samples (Lipson et al., 2018; LeSure-Lester & King, 2004; Liu et al., 2019). For instance, the largest recent study evaluating mental health disparities among college students of color found that Asian American, Latinx, and Multiracial students were more likely to have clinically elevated depression symptoms when each group was compared to all other students. This study also found that Multiracial students experienced elevated anxiety and higher suicide risk relative to all other groups, while African American and Asian American students were less likely to have clinically elevated anxiety (Lipson et al., 2018). Factors contributing to mental health problems among college students of color have also been identified, including experiencing microaggressions, discrimination, imposter syndrome, and negative campus climate (Hwang & Goto, 2008; Nadal et al., 2014; Prelow et al., 2006). However, several prior studies have found no evidence of racial/ethnic differences in mental health concerns among college students (Eisenberg et al., 2007a, 2007b, Mokrue & Acri, 2015). Given the mixed nature of prior findings, additional research is needed to delineate disparities in mental health problems among college students of color. Another limitation of the extant literature is that many studies comprise small groups of racial/ethnic minority students, compared to larger NHW groups (Lipson et al., 2018). In addition, most prior studies have evaluated differences in prevalence of mental health problems, often using established cutoff scores to characterize absence or presence of anxiety and depression (Lipson et al., 2018). Studies that evaluate differences in severity of mental health concerns among diverse students have been less frequently conducted and can be helpful in providing more nuanced clinical information to inform levels of need and treatment allocation. To improve our understanding of mental health needs on the growing number of college campuses that serve largely students of color, samples that reflect the representation of our increasingly diverse college student population are essential.

College campuses have been considered places where disparities in mental health care could be attenuated, because many institutions provide on-campus mental health services and students have relatively high rates of insurance coverage, decreasing practical barriers to care access (McIntosh et al., 2012). However, a multitude of barriers to services for college students of color remain, contributing to persistence of observed racial/ethnic disparities on college campuses (Hunt et al., 2015; Lipson et al., 2018). Commonly endorsed obstacles to mental health treatment for students of color include financial concerns, a lack of time for treatment, a lack of perceived need for treatment, and stigma (Lispon et al., 2018; Miranda et al., 2015). Indeed, many studies illustrate that students of color are less likely to receive mental health services than NHW students, and when they do, they more likely to drop out of treatment early (Hunt et al., 2015; Kearney et al., 2005). In a recent study, 45.5% of NHW students with mental health needs received past-year treatment, compared to only 33% of Latinx, 25% of African American, and 18.9% of Asian American students (Lipson et al., 2018). Persistent inequities in care receipt on college campuses underscore the need to delineate mental health problems and treatment use among college students of color, and implement innovative strategies that can bridge gaps in care for traditionally underserved groups.

### Strategies for Reducing Unmet Need and Disparities

One proposed pathway to enhancing equity in mental health care in college settings is through online screening and digital mental health interventions (DMHI) (Lattie et al., 2019a, 2019b; Muñoz et al., 2010; Schueller et al., 2019). Provision of online mental health screening and treatment is considered an advantageous method for reaching college students in general, many of whom report a lack of time and perceived inconvenience for face-to-face services, but express high levels of comfort and acceptance of technology (Healthy Minds Study, 2019; Lattie et al., 2019a, 2019b). Some research also suggests college students of color in particular report a preference for online screening and interventions, highlighting the unique potential for DMHI to engage populations with historically lower rates of treatment seeking in care (Dunbar et al., 2018; Lungu & Sun, 2016). In addition, online screening and interventions have potential to directly address well-documented barriers to care, such as stigma and lack of time.

To date, there is no evidence to suggest that universal screening for mental health problems can directly result in reductions in racial/ethnic disparities in mental health service use (Guo et al., 2017). Furthermore, no known studies have tested the effect of universal screening on disparity reduction on college campuses. To achieve intended effects on disparity reduction, screening efforts may consider involving providing personalized feedback about self-reported symptom profiles, and stigma can be mitigated by enabling students to access screening and intervention resources in private on personal electronic devices (Yorgason et al., 2008). Though these strategies can promote engagement among all college students, ethnic minority students endorse more treatment barriers and report lower rates of help-seeking and service use relative to NHW students (Eisenberg et al., 2011; Miranda et al., 2015). Thus, online screening and intervention may be a particularly promising avenue to reducing racial disparities in care.

Furthermore, despite their potential to circumvent barriers and increase care access for marginalized groups, the success of DMHI is consistently constrained by limited user uptake and engagement (Lattie et al., 2019a, 2019b; Torous et al., 2018). Studies of DMHI among college students have suffered from low rates of recruitment, pointing to concerns regarding feasibility and acceptability of these programs (Levin et al., 2020). One study found that just 7% of college students reported having used mental health apps, and of these, only 24% continued using the app for four weeks or longer (Kern et al., 2018). Although many have cited the potential of DMHI to reduce disparities, college students of color students remain underrepresented in the literature on digital mental health (Knowles et al., 2014). To our knowledge, there is no evidence about whether these tools can successfully alleviate the disparities frequently observed in traditional care delivery settings (Lattie et al., 2019a, 2019b; Ramos & Chavira, 2019). Additional research is needed to understand diverse student mental health needs and examine whether disparities in DMHI uptake exist when these programs are made available and accessible to students.

The current study aims to address these gaps by evaluating racial/ethnic differences in mental health problems and treatment uptake within the context of a large research and treatment initiative, the University of California, Los Angeles (UCLA) Depression Grand Challenge (DGC) Screening and Treatment for Anxiety and Depression (STAND) program. The overarching goal of the STAND program is to provide comprehensive screening and treatment to students with mental health needs, primarily in domains of depression, anxiety and suicidality. Two research questions are explored in the current study.

1. Are there racial/ethnic differences in mental health problems (depression severity, anxiety severity, suicidality), and reported history of mental health treatment within a diverse sample of students who completed a mental health screening survey?
2. Among students who took the screening survey and were eligible for free, online therapy or face-to-face treatment (depending on severity level), are there racial/ethnic differences in rates of treatment enrollment and treatment initiation?

## Method

Data for this study were drawn from the UCLA DGC STAND research initiative (https://www.stand.ucla.edu/). UCLA is a large, public university serving a racially/ethnically diverse student population. The STAND program involves two core components within the scope of a series of research studies: screening and treatment. First, all registered UCLA students were eligible to complete an online screening survey including demographics and assessment of mental health symptoms (described in detail below under “Screening”). After students completed the screener, they were provided with information about their mental health symptoms and if eligible, were offered free mental health treatment corresponding to their symptom level within a four-tiered treatment design. Those with no depression or anxiety were offered behavioral health tracking only (Tier 0). Those with mild depression or anxiety were offered a 6-module internet-based cognitive behavioral therapy (iCBT) program with certified peer support in adjunctive coaching sessions (Tier 1). Those with moderate depression or moderate to severe anxiety were offered the same iCBT program with an advanced certified peer support (Tier 2). Those with severe depression or suicide risk were offered face-to-face gold standard evidence-based treatment, tailored to their presenting needs, within the STAND program's Innovative Treatment Clinic (ITN). Face-to-face services included evidence-based psychotherapy with or without pharmacotherapy, provided by clinical psychology graduate students or postdoctoral fellows (supervised by licensed clinical psychologists), and psychiatry residents (supervised by attending psychiatrists). Treatment was provided for up to 10 months, with an average length of ~ 12 weeks. All research procedures were approved by the UCLA Institutional Review Board.

### Participants

**Fig. 1:**
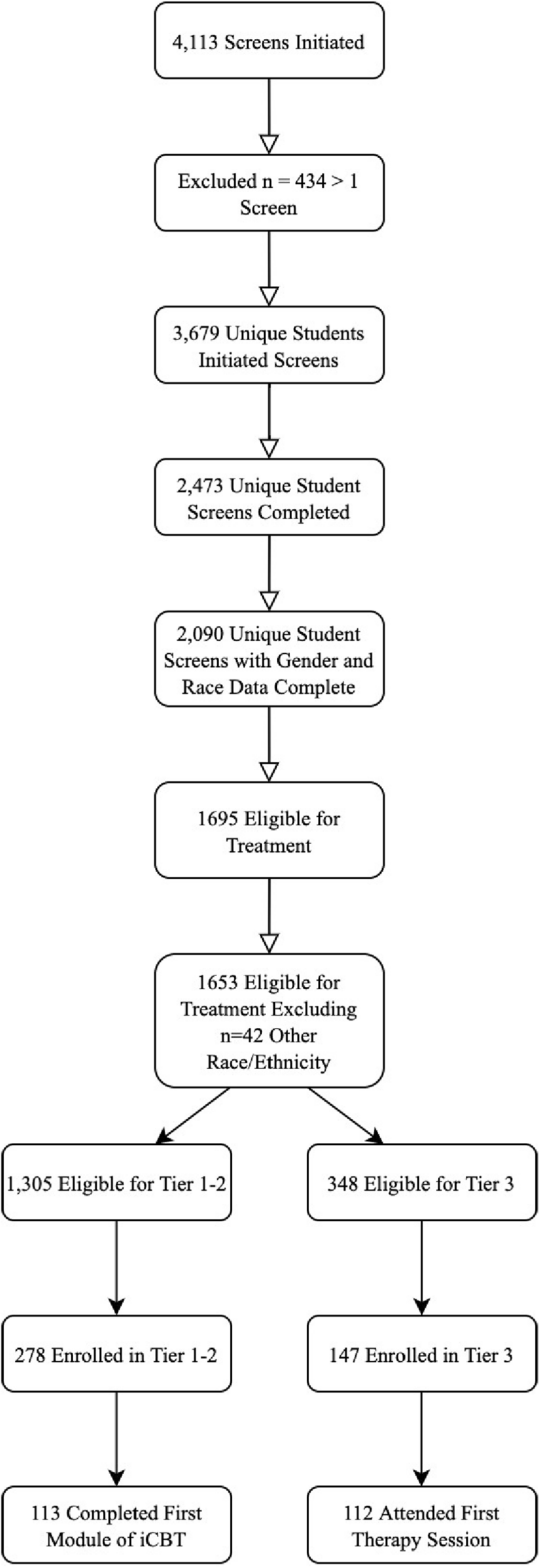
Consort flow from screening to initial use for study sample

Eligible participants for the screening survey included all registered, matriculating UCLA students ages 18–65 with English proficiency. Data in the present study are drawn from the period April 2018–February 2020. This timeframe was selected because demographic information on student race/ethnicity was collected within the screening survey within this period. Students were invited to participate in the screening through a number of methods within a campaign to raise awareness about depression, including through print/online flyers distributed at various campus locations, advertising on social media, emails sent out from the Registrar’s office and campus departments, recruitment messages on UCLA websites, and STAND program staff participation in campus events. Figure 1 displays the consort flow that illustrates the derivation process for the current study sample. Within the study timeframe, a total of 4113 screening surveys were initiated, with 434 students taking the survey more than once. For these n = 434 students, a rule was created by which their first screening encounter was used, unless they enrolled in treatment at subsequent screening encounter, in which case that encounter was used in the current study. Of the 3679 unique screens, 2473 students (67.2%) completed the entire screener. Chi- square analyses indicated no racial/ethnic differences in screener initiation vs. completion χ^2^ = 6.91, df = 1, p = 0.23. Of the 2473 students who completed the screener, a total of 383 were excluded due to missing data on race/ethnicity. Thus, final screening sample size of participants in the present study was n = 2090.

#### Screening Procedures

Students first completed a number of eligibility questions (e.g., age, English fluency), items assessing history of mental health diagnoses and treatment, and demographic questions (race/ethnicity, sex, gender identity). Next, students completed the Computerized Adaptive Test–Mental Health (CAT-MH) (Gibbons et al., 2012, 2013, 2014), an adaptive questionnaire designed to rapidly and reliably assess mental health symptoms in domains of depression, anxiety, suicidality and others (described in measures section). The CAT-MH has consistently demonstrated high validity and reliability across a multitude of settings (Gibbons & DeGruy, 2019). Students were allowed to take the mental health screening survey an unlimited number of times, at least two weeks apart.

#### Treatment Procedures

After completing the screening survey, students were directed to a webpage that provided them with personalized feedback on their symptoms and information about treatment tier eligibility. Students eligible for Tiers 1 and 2 (mild to moderate depression, or mild to severe anxiety) were provided with a link that enabled them to review the consent form for the online therapy study, enroll in the treatment study immediately, and sign up for an orientation led by a certified peer coach. Simultaneously, eligible students also received an email with a link to schedule this orientation. If no orientation was scheduled two days after completing screening, study team members began contacting the participant to encourage them to schedule an orientation. Participants were contacted up to three times over a two week period, via text messaging or email, depending on their indicated preference. These messages were pre-scripted and included a short, encouraging message with a link to schedule the orientation. If no orientation was scheduled after three contacts over two weeks, contact was ceased. Participants who attended orientations were then provided with an account and login permissions to the internet-based cognitive behavioral therapy, This Way Up (TWU; Newby et al., 2013, 2014). The TWU Mixed Anxiety and Depression Course was utilized in this study, and is comprised of six online modules, and participants were allotted 8 weeks to complete all modules. Participants in Tier 1 and 2 were offered support from peer coaches, provided via 30-min weekly coaching sessions intended to review application of module content, troubleshoot and provide motivational support. Students who opted into coaching (78.3%) were assigned a coach and scheduled for their first meeting with their coach during the orientation visit.

Students eligible for Tier 3 face-to-face treatment (severe depression or suicide risk) were contacted via telephone by a member of the research team to invite them to participate in the Tier 3 face-to-face treatment study within 24 h after completing screening. Eligible students who did not respond to the outreach call were contacted up to three times over a two week period, by text message or email depending on their preference. Similar to Tiers 1–2, these outreach efforts consisted of encouraging messages and provided the office phone number. Additional inclusion criteria were evaluated at this phone call, including: internet access, agreement to establish long-term care with an external provider if indicated after Tier 3 treatment ended, willingness to install an app to monitor activity and behavior, agreement to participate in research study procedures including symptom assessments and blood draws, and proficiency in English. Exclusion criteria were also evaluated at this phone call and included: unstable suicidality, current substance abuse interfering with treatment, primary diagnosis of psychosis unrelated to depression, neurological conditions, severe uncontrolled medical conditions, cognitive impairment, and current treatment by psychologist/psychiatrist that would not be discontinued for the course of Tier 3 treatment. Eligible participants were scheduled for a baseline visit to complete a variety of research assessments and an evaluation by a clinical psychology assessor. Following this, participants were assigned a clinician and weekly, face-to-face treatment began.

### Measures

#### Race/Ethnicity

Students responded to a question identifying their racial background in the screening survey (“What race to you consider yourself to be?”). They were provided a list of 19 responses in checkbox format consistent with the UC Registrar item (multiple selections were allowed). Students also responded to the question “Do you consider yourself to be Hispanic/Latino?” (yes/no). A single race/ethnicity variable was created with the following mutually exclusive racial/ethnic groups: NHW, Black/African American, Asian/Asian American, Hispanic/Latino (referred to hereafter as Latinx), Multiracial (included all those who selected more than one race, and those who identified ethnically as Hispanic and any race/ethnicity other than white), and Other (included all those who identified as belonging to another racial/ethnic group not listed on the questionnaire, in addition to those who identified as Native Hawaiian/Pacific Islander (n = 2) and Native American/Alaska Native (n = 6)).

#### Depression

Depression was measured using the Computerized Adaptive Test–Depression Inventory (CAT-DI) (Gibbons et al., 2017), which assesses several domains of depression, including mood, cognition, behavior, somatic problems, and suicidal ideation. The total item bank consisted of 389 items, and a mean of 12 items were administered per participant in the validation study (Gibbons et al., 2017). Each participant received a CAT-DI score ranging from 0–100, with 0–49 indicating minimal depression, 50–65 mild depression, 66–75 moderate depression, and 76–100 severe depression. These cut-points were empirically derived based on a mixture of normal distributions (Gibbons et al., 2012).

#### Anxiety

Anxiety was assessed with the Computerized Adaptive Test–Anxiety (CAT-ANX) (Gibbons et al., 2014). The full item bank consisted of 467 items, with an average of 12 items administered per participant in the validation study (Gibbons et al., 2014). Similar to depression, domains of anxiety assessed included mood, behavior, cognition and somatization. CAT-ANX scores ranged from 0–100, with scores of 0–34 indicating minimal anxiety, 35–49 mild anxiety, 50–64 moderate anxiety, and 65–100 severe anxiety. Similar to the CAT-DI, cut points were empirically derived by transforming scores from normal distributions (Gibbons et al., 2014).

#### Suicidality

The current study utilized a variable characterizing positive suicide screen based on responses to four items administered within the Computerized Adaptive Test–Suicide Scale (CAT-SS) (Gibbons et al., 2017). Three items assessed for presence of past month suicidal ideation, intent, and plan. One item assessed for suicidal behavior over the past three months (including attempt, aborted or interrupted attempt, and preparatory acts). Students who endorsed past-month suicidal ideation with intent or plan, or past three-month suicidal behavior were considered positive suicide screens and received outreach from the study team following a standardized risk assessment protocol. Other items from the CAT-SS have been utilized in the STAND program, but were not used in the current study.

#### Enrollment

Enrollment occurred once a student consented to and enrolled in the treatment study for which they were eligible after screening. For the purpose of this study, enrollment was characterized by a dichotomous variable indicating whether or not the participant was assigned a treatment study identifier.

#### Initial Treatment Use

Initial treatment use was measured with a dichotomous variable identifying whether or not the participant completed the first online therapy module (for Tiers 1–2) or attended an initial therapy session (for Tier 3).

These two dichotomous items were also combined to create a categorical variable of engagement, characterizing whether the participant was eligible but did not enroll (0), enrolled but did not participate in an initial treatment session, (1) or enrolled and participated in an initial treatment session (2).

#### Data Analytic Plan

Data were collected using REDCap (Harris et al., 2019) Statistical analyses were conducted using Stata Statistical Software—Version 14. To examine our first research question (racial/ethnic differences in depression severity, anxiety severity, suicidality and prior treatment receipt in screening sample), two ordinal logistic regression models examined whether membership in each of the racial/ethnic minority groups was associated with differential odds of falling into a more severe category of (1) depression or (2) anxiety relative to NHW students. Ordinal logistic regression models were selected to optimize clinically meaningful interpretation of differences, given that categorical cutoffs have been empirically established for the CAT-MH (cutoffs described above). Next, binary logistic regression models were used to specify the effect of racial/ethnic group on (1) the positive suicide screen outcome, (2) prior mental health treatment receipt outcome, and (3) treatment eligibility for any treatment tier within the STAND program. All models utilized simple contrasts to compare each racial/ethnic minority group to NHW students.

To assess the second research question, binary logistic regression models were employed to evaluate the effect of race/ethnicity on initial treatment use among those who screened eligible for treatment. Three models were specified for the initial use outcome (1) across all tiers, (2) within Tiers 1–2 (online therapy) and (3) within Tier 3 (face-to-face therapy). To evaluate racial/ethnic disparities across stages (e.g. eligibility vs. enrollment, enrollment vs. initial use) a multinomial logistic regression model was used to assess the effect of race/ethnicity on a categorical treatment engagement variable. This outcome was created with values of 0 = eligible but did not enroll, 1 = enrolled but did not complete an initial treatment session, and 2 = enrolled and completed an initial session (either in person for Tier 3, or online for Tiers 1–2). The reference outcome utilized in the model was 1 = enrolled. Depression, anxiety, treatment tier eligibility (Tiers 1–2 vs. Tier 3), gender (female vs. male) were entered as covariates in the model.

## Results

### Descriptive Statistics

Table 1 displays the screening sample study composition compared to the university student body demographics. Inferential tests comparing the screening sample demographics to the full student body breakdown were not conducted because our screener race/ethnicity item did not differentiate between international vs. domestic students.

**Table 1:** Descriptive data on race/ethnicity and gender in screening sample and student body

Demographic variable	Screening sample (n = 2090)	Full student body (n = 45,930) (%)
Race/Ethnicity		
Asian/Asian American	34.6%	24.9
White	26.7%	28.6
Latinx	23.2%	18.9
Multiracial	08.5%	05.4
Black/African American	04.4%	03.0
Other	02.5%	03.0
International students	–	16.3
Gender		
Female	72.7%	54.6
Male	27.3%	45.4

International vs. domestic, and graduate vs. undergraduate student status data were not collected in mental health screening survey

### Racial/Ethnic Differences in Mental Health Problems and Treatment History

#### Depression

**Fig. 2:**
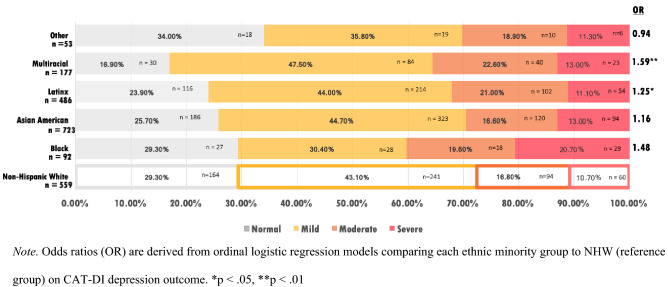
Depression level by race/ethnicity

An ordinal logistic regression on the categorical outcome of depression revealed that Latinx (OR 1.25, CI 1.00–1.57, p < 0.05), and Multiracial (OR 1.59, 1.18–2.16, p < 0.01) students were significantly more likely to be in a more severe depression category, relative to NHW students, and Black/African American students were marginally significantly more likely to be in a more severe depression category (OR 1.48, CI 0.97–2.26, p < 0.10). Figure 2 depicts the rates of each level of depression for each racial/ethnic group.

#### Anxiety

**Fig. 3:**
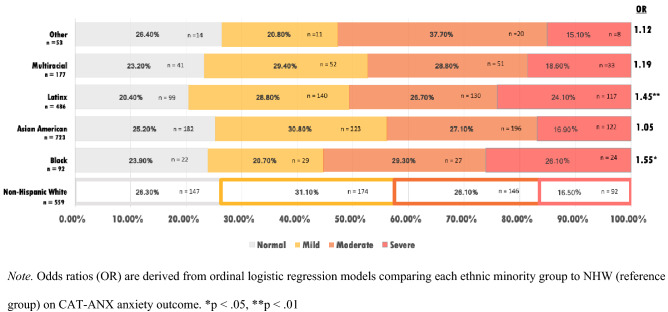
Anxiety level by race/ethnicity

For the categorical anxiety outcome, an ordinal logistic regression revealed that Black/African American students (OR 1.55, CI 0.88–2.15, p < 0.05), and Latinx students (OR 1.45, CI 1.16–1.80, p < 0.01) were significantly more likely to be in a more severe anxiety category, compared to NHW students. Figure 3 depicts these rates for each racial/ethnic group.

#### Suicidality

**Fig. 4:**
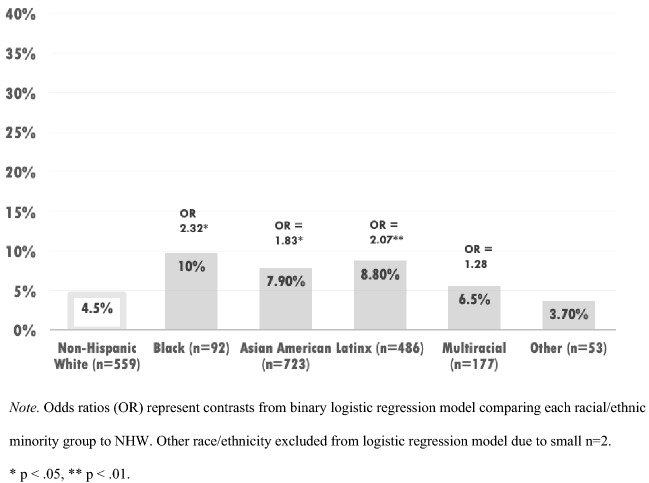
Positive suicide screen by race/ethnicity

For the outcome of positive suicide screen, a binary logistic regression found Black/African American (OR 2.31, CI 1.04–5.13, p < 0.05), Asian American (OR 1.83, CI 1.13–2.97, p < 0.05), and Latinx (OR 2.07, CI 1.25–3.45, p < 0.01) students were significantly more likely to screen positive for suicide risk relative to NHW students (see Fig. 4).

#### Prior Treatment Receipt

**Fig. 5:**
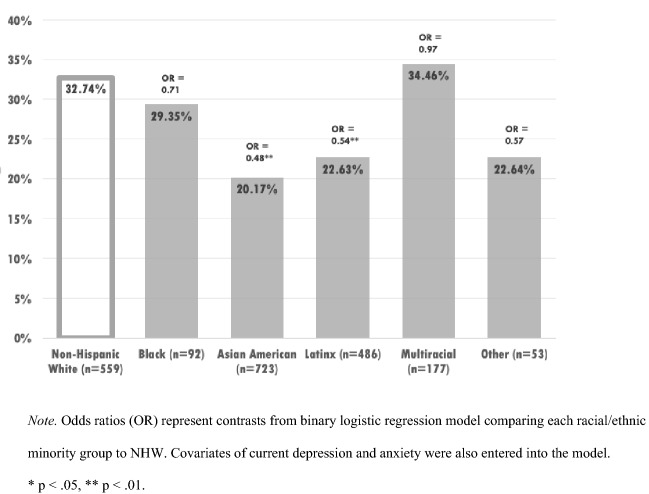
Prior mental health treatment or diagnosis receipt by race/ethnicity

Differences also emerged for likelihood of prior self-reported mental health treatment or diagnosis receipt, such that Asian American (OR 0.48, CI 0.37–0.62, p < 0.001), and Latinx (OR 0.53, CI 0.40–0.70, p < 0.001) students were significantly less likely to report having received previous treatment or diagnosis, compared to NHW students, covarying for the effects of current depression and anxiety severity (Fig. 5). Covariates of depression (OR 1.02, CI 1.01–1.02, p < 0.001) and anxiety (OR 1.02, CI 1.01–1.02, p < 0.001) were also statistically significant predictors in the model.

### Racial/Ethnic Differences in Treatment Enrollment and Initiation

Percentages of penetration by levels of engagement in treatment (eligibility, enrollment and initial use) by race/ethnicity are presented in Table 2. Results indicated no statistically significant racial/ethnic or gender differences in initial use of treatment across tiers. The effect of treatment tier eligibility was significant, such that those who were eligible for Tier 3 were more likely to initiate use of treatment than those eligible for Tiers 1–2 (OR 5.23, CI 3.52–7.78 p < 0.001). The effect of anxiety severity was also significant, such that those with higher anxiety severity scores were more likely to initiate treatment across tiers (OR 1.02, CI 1.00–1.03, p < 0.01).

**Table 2:** Descriptive data on eligibility, enrollment and by race/ethnicity

	NHW n = 559	Black n = 92	Asian Am n = 723	Latinx n = 486	Multiracial n = 177	Total n = 2037
All Tiers						
Eligible	441 (78.9%)	74 (80.4%)	574 (79.4%)	406 (83.5%)	158 (89.3%)	1653 (81.1%)
Enrolled	105 (23.8%)	20 (27.0%)	163 (28.4%)	98 (24.3%)	39 (24.7%)	425 (25.7%)
Initial use	60 (13.6%)	8 (10.8%)	83 (14.5%)	54 (13.3%)	20 (12.7%)	225 (13.6%)
Tiers 1 and 2						
Eligible	365 (65.3%)	52 (56.5%)	436 (60.3%)	322 (66.3%)	130 (73.4%)	1305 (64.1%)
Enrolled	71 (19.4%)	13 (25.0%)	102 (23.4%)	67 (20.8%)	25 (19.2%)	278 (21.3%)
Initial use	33 (9.0%)	3 (5.8%)	37 (8.5%)	31 (9.6%)	9 (6.9%)	113 (8.6%)
Tier 3						
Eligible	76 (13.6%)	22 (23.9%)	138 (19.1%)	84 (17.3%)	28 (15.8%)	348 (17.1%)
Enrolled	34 (44.7%)	7 (31.8%)	61 (44.2%)	31 (36.9%)	14 (50.0%)	147 (42.2%)
Initial Use	27 (35.5%)	5 (22.7%)	46 (33.3%)	23 (27.4%)	11 (39.3%)	112 (32.2%)

Other ethnic minority race/ethnicity (n = 42) excluded from engagement analyses due to n < 5 within initial use level of DV. Row percentages reflect the % of students who were eligible among those screened, % of students who enrolled among those who were eligible; % of those with initial use among those who were eligible

**Fig. 6:**
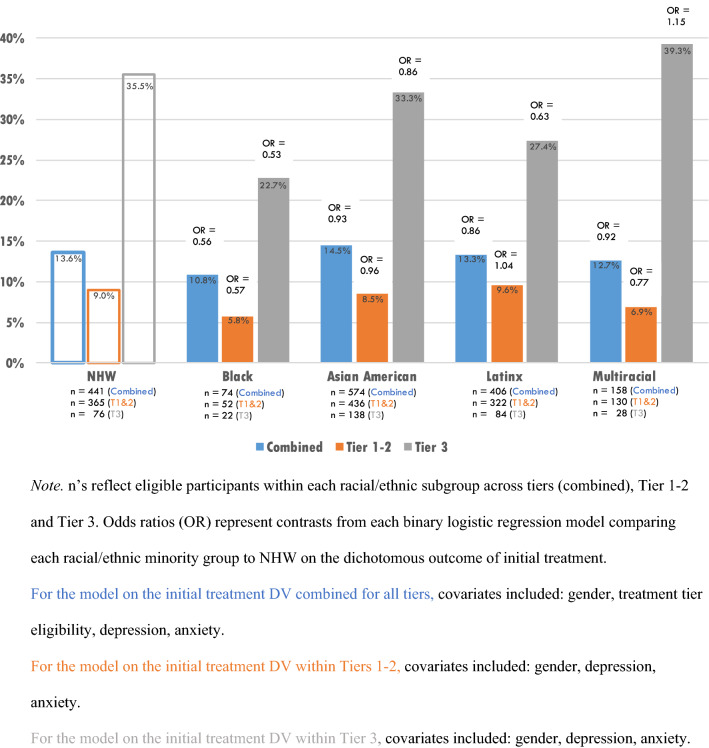
Initial use of treatment by race/ethnicity. n’s reflect eligible participants within each racial/ethnic subgroup across tiers (combined), Tier 1–2 and Tier 3

Two additional binary logistic regression models were specified to explore racial/ethnic differences in initial treatment use within each tier. Models were considered exploratory due to small sample sizes for initial use for Black and Multiracial students when subset by tier. For the model within Tiers 1–2, covariates of depression, anxiety, and gender were entered. Results indicated no statistically significant racial/ethnic differences or effects for gender or depression on initial use of Tier 1–2 treatment. The effect of anxiety was significant, such that those with increased anxiety were more likely to engage in initial use of Tier 1–2 treatment (OR 1.02, CI 1.01–1.04, p < 0.01). For the Tier 3 model, no significant racial/ethnic differences, or effects for gender, anxiety or depression were found were found. Table 3 shows model results, and Fig. 6 displays rates of initial use by race/ethnicity and tier.

**Table 3:** Binary logistic regression on initial use of treatment

Variable	Initial use combined tiers		Initial use tiers 1–2		Initial use tier 3	
	OR (95% CI)	p value	OR (95% CI)	p value	OR (95% CI)	p value
Race/Ethnicity (NHW)						
Black/African American	0.56 (0.25–1.27)	0.163	0.57 (0.17–1.95)	0.370	0.53 (0.18–1.62)	0.268
Asian American	0.93 (0.64–1.35)	0.698	0.96 (0.59–1.58)	0.875	0.86 (0.48–1.56)	0.621
Latinx	0.86 (0.57–1.31)	0.487	1.04 (0.62–1.74)	0.894	0.63 (0.32–1.26)	0.193
Multiracial	0.92 (0.52–1.62)	0.762	0.77 (0.36–1.68)	0.518	1.15 (0.47–2.84)	0.748
Male (Female)	0.96 (0.69–1.35)	0.840	1.01 (0.64–1.59)	0.961	0.92 (0.56–1.61)	0.737
Tier 3 Eligibility (Tier 1–2)	5.23 (3.52–7.78)***	< 0.0001	–	–	–	–
Depression Severity	0.98 (0.97–1.00)	0.083	0.99 (0.97–1.01)	0.219	0.98 (0.96–1.01)	0.225
Anxiety Severity	1.02 (1.00–1.03)**	0.004	1.02 (1.01–1.04)**	0.005	1.01 (0.99–1.03)	0.237

Reference group is parenthesized for categorical IVs

*p < 0.05, **p < 0.01, ***p < 0.001

Results from an exploratory multinomial logistic regression model examining possible racial/ethnic differences at each step of the initial engagement process (i.e. eligibility vs. enrollment, enrollment vs. initial use) indicated that the relative risk of being eligible but not enrolling in treatment was significantly lower for Asian American students compared to NHW students (RRR 0.67, CI 0.45–0.99, p < 0.05). In addition, the effect of anxiety was significant, such that the relative risk of being eligible for treatment but not enrolling was lower for those with higher levels of anxiety (RRR 0.97, CI 0.97–0.99, p < 0.001). The relative risk of being in the initial treatment category vs. being enrolled without initiating care, was significantly higher for students in Tier 3, compared to those in Tiers 1–2 (RRR 6.41, CI 3.58–11.47, p < 0.001) (Table 4). There were no significant racial/ethnic differences in relative risk of initiating treatment vs. enrolling but not initiating.

**Table 4:** Multinomial logistic regression on engagement in treatment outcome

Variable	Relative risk of not enrolling when eligible (Eligible vs. Enrolled)		Relative risk of initiating treatment when enrolled (Initial Use vs. Enrolled)	
	RRR (95% CI)	p value	RRR (95% CI)	p value
Race/Ethnicity (NHW)				
Black/African American	0.66 (0.32–1.35)	0.260	0.40 (0.15–1.08)	0.071
Asian American	0.67 (0.45–0.99)*	0.049	0.66 (0.40–1.10)	0.109
Latinx	0.99 (0.63–1.55)	0.967	0.86 (0.48–1.51)	0.591
Multiracial	0.83 (0.46–1.48)	0.530	0.78 (0.37–1.66)	0.519
Male (Female)	0.89 (0.63–1.26)	0.526	0.87 (0.56–1.37)	0.572
Tier 3 Eligibility (Tier 1–2)	1.26 (0.77–2.07)	0.347	6.41 (3.58–11.47)***	< 0.001
Depression severity	1.00 (.98–1.02)	0.743	0.99 (0.97–1.01)	0.288
Anxiety severity	0.97 (0.97–0.99)***	< 0.001	1.00 (0.98–1.01)	0.590

Reference group is parenthesized for categorical IVs. The base outcome of enrolled but did not initiate use (1) was compared to the outcomes of eligible but did not enroll (0) and enrolled and initial use (2)

*p < 0.05, **p < 0.01, ***p < 0.001

## Discussion

The current study provides preliminary evidence on racial/ethnic differences in mental health problems, enrollment and initial use of digital and in-person treatment among students who self-selected into a mental health screening and treatment study at a diverse, public four-year university serving a large proportion of students of color. First, the significant representation of students of color in our sample (73.3%) suggests online screening may be an effective tool for reaching students from historically underserved groups on campus. We also identified racial/ethnic variation in levels of depression, anxiety and suicidality among students screened. Latinx and Multiracial students were more likely fall into a more severe depression level relative to NHW students, Black/African American and Latinx students were more likely to fall into a more severe anxiety level relative to NHW students, and Asian American, Black/African American and Latinx students were more likely to screen positive for suicide risk compared to NHW students. Although students of color were less likely to have received prior treatment, they were no less likely than NHW students to enroll and initiate treatment in this program. Findings suggested that eligible Asian American students were significantly more likely to enroll in treatment relative to eligible NHW students, and there were no racial/ethnic disparities between NHW students and racial/ethnic minority students in initial treatment use.

Overall, rates of mental health prevalence in this screening sample fell within the range of previous estimates. 30.6% of students screened fell within the moderate-severe range for depression, which is within the range of depression prevalence estimates in recent large-scale studies of college students (16.8% to 41.1%; Duffy et al., 2019; Lipson et al., 2018). In our sample, 46.2% of students had moderate to severe anxiety, which falls within the range identified in prior studies (17.1% to 63.3%; Duffy et al., 2019; Lipson et al., 2018). Moreover, 7.0% of our screening sample screened positive for suicide risk, comparable to other study prevalence rates for past year suicidal ideation among college students (Lipson et al., 2018). Importantly, because the students in our study represent a self-selected sample, these mental health problem and severity rates do not represent a direct comparison to study samples in which students are randomly selected and screened. Yet, our findings suggest significant need among students screened, with 81.1% of screened students eligible for some level of treatment, which aligns with findings of previous studies showing high levels of need (Lipson et al., 2018).

Our interrogation of racial/ethnic differences in mental health problems in the screened sample revealed that students from some ethnic minority groups were at elevated risk for experiencing more severe depression and anxiety and elevated suicide risk compared to NHW students. These differences in mental health problems may point to the need for mental health service systems to reduce barriers to engaging ethnic minority students in care. However, this finding must be interpreted with caution, given study design limitations. Because students self-selected into screening, as opposed a universal screening, our findings may not reflect overall higher need among ethnic minority students on campus. An alternative explanation for the elevated severity observed among ethnic minority students screened may be that proportionally more ethnic minority students with mental health need opted to take the screener compared to NHW students. This interpretation may be less plausible given that the penetration of screening among students of color appeared higher than among NHW students, but it cannot be ruled out. We can conclude that among ethnic minority students who opted into mental health screening, there was a higher base rate of demonstrated need for care, as compared to NHW students.

In general, these findings align with prior evidence that found elevated mental health concerns among students of color (Lipson et al., 2018). Although the current study did not assess determinants of mental health outcomes that might explain elevated severity among college students of color, a multitude of social and structural determinants of mental health disparities are well established. For example, experiencing discrimination and racial microaggressions on campus have been consistently associated with poorer mental health outcomes, including depression, anxiety and suicide risk, among students of color (Hwang & Goto, 2008; Nadal et al., 2014; Prelow et al., 2006). Perceived discrimination has also been associated with lower perceptions of social support, which in turn has been linked with depression among Black/African American college students (Prelow et al., 2006). Imposter feelings experienced by Asian American, Black/African American, and Latinx college students have been found to moderate and mediate links between perceived discrimination and depression and anxiety symptoms (Cokley et al., 2017). Negative experiences of campus climate and lower feelings of belongingness have also been linked with poorer mental health outcomes among college students of color and first-generation college students (Arbona & Jimenez, 2014; Stebleton et al, 2014). Thus, several potential explanations for the elevated rates of mental health problems among college students of color observed in this screening study and in prior studies exist. Studies that continue to explore and identify social determinants of racial disparities among college students represent a key direction for future research.

Our findings also suggested that Asian American and Latinx students screened were less likely than NHW students to have received previous mental health treatment, covarying for current depression and anxiety (Lipson et al., 2018; Liu et al., 2019). In our sample, 20.2% of Asian American and 22.6% of Latinx students reported that they have received prior treatment or a diagnosis, compared to 32.7% of NHW students. These findings are aligned with prior research that underscores enduring disparities in mental health service use on college campuses (Lipson et al., Liu et al., 2019). To mitigate these disparities, numerous strategies have been employed, such as community outreach, gatekeeper training and culturally tailored programs and messaging (Banks, 2020; Boone et al., 2011; Lipson et al., 2018). Digital mental health tools have also been widely considered as a viable format for narrowing gaps in care for college students (Lattie et al., 2019a, 2019b).

Although online screening and interventions have long been considered a promising strategy to reduce disparities, no studies to our knowledge have empirically supported this claim in college student populations. Our findings indicated students of color were no less likely than NHW students to enroll in or initiate treatment offered through STAND. This finding sits counter to the research on mental health service use at large, which consistently highlights that students of color are less likely to receive mental health care compared to NHW students (Eisenberg et al., 2011; Herman et al., 2011). The current study results provide support for the utility of digital mental health tools to mitigate disparities in utilization of care. Thus, campus outreach and messaging about the important goal of reducing the burden of depression, in conjunction with online mental health screening, may be an effective avenue to reaching underserved students to engage them in mental health monitoring and pathways to care.

Although this study did not find racial/ethnic disparities in enrollment and initial engagement with treatment, key differences in rates of engagement among those allocated to web-based therapy and face-to-face treatment were apparent. Students who were eligible for face-to-face treatment were significantly more likely to initiate treatment than those eligible for online therapy. This finding may be explained by differences in severity. Exemplified by their screening into a higher tier, the elevated severity experienced by these students likely confers increased functional impairment and subjective distress that drive greater perceived need for treatment than those with mild and moderate symptoms. Further, those who screened into face-to-face treatment were contacted multiple times by a study team member to recruit them into the study and enroll them, as opposed to being provided with an online link which required students to scroll through multiple screens and attend a virtual or in-person orientation visit in order to enroll in online therapy. The greater investment in converting screening to enrollment through human contact, compounded with increased symptom severity and impairment, are likely factors influencing the higher rate of engagement observed for face-to-face therapy. In contrast, user burden associated with requirements to move through several webpages with discrete steps in order to initiate online therapy in tiers 1 and 2 may have constrained rates of uptake. Indeed, usability of digital mental health tools has been found to be a key factor associated with engagement (Ng et al., 2019), emphasizing the need for streamlined and user-friendly processes to enhance engagement in digital mental health innovations.

Just over 1 in 5 students who were eligible and offered free online therapy enrolled in treatment, and 8.7% initiated use of treatment. Few prior studies have reported data on uptake and usage, complicating the relative assessment of this success rate. In a systematic review of digital mental health interventions in college students, Lattie et al. found that only 8 of 81 studies reported data on uptake, and rates of enrollment in these 8 studies ranged from 1 to 37% (Lattie et al., 2019a, 2019b). Though the enrollment rate in the online therapy program found in the present study falls within this range, many observers would agree that these rates of enrollment and initial use are suboptimal, because the large majority of students who are eligible for and offered care do not enroll and initiate use. Given that offering free, online care already significantly reduces many barriers, including cost and inconvenience, attention to additional factors that influence program uptake is needed, and innovative strategies that improve their initial use must be implemented.

Even among students who opted to enroll in online therapy, fewer than half completed their first online therapy session. Other studies have similarly found low rates of program initiation among individuals who enroll in online intervention programs (Arean et al., 2016; Bedford et al., 2018). These results are contextualized by evidence noting a substantial gap between human intention and behavior (Webb & Sheeran, 2006). A myriad of factors have been found to influence engagement with digital tools, including factors related to the program user (e.g. perceived relevance, motivation, self-efficacy) characteristics of the program itself (e.g. design features, ease of use), and features of the context the program is implemented in (e.g. integration within service system, accessibility, cost), but the centrality of each of these variables in relation to initial use of programs is not well understood (Perski et al., 2017). Nonetheless, is it likely that commonly observed barriers to digital therapy engagement were at play in the present study, including factors such as limited perceived usefulness and fit, technical issues, limitations with regard to personalization and customizability, concerns about privacy and confidentiality, and limited integration of the program with user daily life (Borghouts et al., 2021). Digital therapy programs that explicitly target these engagement barriers can improve uptake and retention in care. Given the current study rates of uptake and the well-established literature on barriers to engagement, we have designed STAND Digital Therapy, a modular program that draws upon existing evidence-based interventions to target a range of disorders. To directly address issues related to engagement, this program utilizes measurement-based care to guide the selection and tailoring of personalized treatment packages that address specific mental health concerns reported by the individual. The program’s personalized packages were designed to maximize engagement, user friendliness, and interactivity, with an emphasis on diversity and inclusion. Research directions that focus on improving uptake of digital mental health programs are also essential to expand the reach of these interventions to currently underserved students.

A number of limitations must be considered in the interpretation of study findings. As previously noted, though STAND offered screening to all UCLA students, it is likely that those who self-selected into taking the screener had elevated interest or concerns about their mental health, as the program was advertised as a mental health tracking, screening and treatment resource. Given significant variability in mental health problems observed across campuses, the results of this study should be interpreted bearing this knowledge in mind (Eisenberg et al., 2013). Further, the sample of Black/African American, Multiracial and Other ethnic minority students in our sample was relatively small, limiting power to detect differences for these groups. In particular, for models comparing initial treatment use for NHW vs. Black students, parity in treatment use cannot be assumed, given potential for insufficient power to detect effects due to the small number of Black/African American students who initiated treatment use across tiers. Furthermore, we recognize that within the broad categories of each racial/ethnic group in this study, there are many subgroups with cultural differences, distinct histories and migration patterns. Our findings do not delineate unique differences between these subgroups. Future studies should describe the subgroups within these monolithic racial/ethnic categories, in order to foster a deeper and more nuanced understanding of diverse student community needs.

In addition, although our study demonstrated that students of color were well represented in an online screening sample, we were not able to compare the representation of each racial/ethnic group in our sample to the overall student body demographics using inferential tests, because we did not attain data on international student status in our screening survey. Given that the university serves a significant proportion of international students, conclusions regarding penetration of our screening tool among these students cannot be drawn. International students represent a large and growing contingent of the U.S. college student population and increased attention to their mental health needs is warranted as a future research direction. This is particularly critical, because evidence to date suggests significant disparities in mental health concerns and help-seeking exist for international students. For example, data indicate that international students are at elevated risk for mental health problems such as depression and anxiety (Cheung, 2011, Han et al., 2013; Wei et al., 2007), but are less likely to utilize mental health services relative to their domestic student counterparts (Clough et al., 2019; Eisenberg et al., 2007a, 2007b; Skromanis et al., 2018). In addition, differences in help-seeking between international and domestic students from the same racial/ethnic group are apparent. In a U.S. sample of Asian American and Asian international college students, international status was related to lower rates of help-seeking, and the association between perceived mental health stigma and personal stigma was stronger for Asian international students compared to Asian American domestic students (Maeshima & Parent, 2020). These findings emphasize the need to delineate differences in treatment and help-seeking between domestic and international students, including among those who share racial/ethnic identities, to enhance our understanding of needs and outreach strategies to diverse student groups.

Last, we do not have data on whether students who did not enroll in STAND treatment were receiving mental health services elsewhere. However, our data suggest that 3 of 4 students who were screened denied any prior history of mental health service receipt, indicating that many students with current needs likely remain underserved. While our study focused on documenting racial/ethnic differences in symptoms and engagement, we did not examine other important predictors of mental health, enrollment and engagement. Given the small number of studies on uptake of digital mental health programs, future research should focus on the unique predictors of online therapy enrollment among college students and identify strategies to promote initial engagement. Although our study did not find racial/ethnic disparities in treatment uptake, our sample was comprised primarily of students of color, highlighting that there continue to be a large proportion of ethnic minority students with mental health needs that remain unmet. Thus, efforts to improve engagement in DMHI should attend to factors that influence help-seeking and treatment receipt within these populations.

Notwithstanding these limitations, this study makes several novel contributions. With regard to enumeration of mental health problem severity, our study is unique in that it contains a large sample of Asian American and Latinx students, who have been traditionally excluded, or included in small sample sizes, in the college mental health literature. While prior samples are comprised of primarily NHW students, ranging from 72 to 74% in recent largescale studies (Duffy et al., 2019), our sample identified primarily as students of color, with just 26.7% of students identifying as NHW. Furthermore, the extant literature largely describes differences in mental health prevalence across racial/ethnic groups, while the current study emphasizes differences in severity of common mental health concerns. From a resource allocation perspective, delineating levels of need of racial/ethnic groups in various contexts is essential to informing design and implementation of student mental health care systems. Additionally, studies that collect data on intersecting identities within racial/ethnic minority samples (e.g. nationality, generation status, religion, gender identity, sexual orientation) and explore levels of mental health need within these subpopulations are needed to advance our knowledge about diverse student needs.

Most notably, our study provides preliminary support for the utility and effectiveness of online mental health tools in reducing disparities in screening and treatment engagement, even among students who have not had prior experiences with mental health services. Although many scholars have implicated the role of digital innovations in mitigating mental health disparities, few studies have empirically assessed the validity of this claim, and evidence within college student populations in especially lacking. The current study findings provide preliminary support for an innovative, comprehensive, online screening and treatment strategy to enhance equity in mental health care for students of color. Aligned with our findings that online screening and treatment have potential to enhance mental health equity among college students, the development and implementation of innovative strategies that enhance uptake and engagement in digital mental health tools represents an essential next step. Our study emphasizes that two windows of time, between eligibility confirmation for treatment and enrollment, and between enrollment and initial use, represent key points of intervention for enhancing engagement. Digital mental health tools hold clear promise for advancing mental health equity among college students, but in order to succeed in this task, we must focus our efforts on better understanding and enhancing student engagement with them.
